# Automated data collection in cancer care: State of play among registries in the United Kingdom and Europe

**DOI:** 10.1177/18333583251378962

**Published:** 2025-10-07

**Authors:** Manuela Roman, Stephen Ali, Nader Ibrahim, Thomas D Dobbs, Hayley Hutchings, Iain S Whitaker

**Affiliations:** 1 Swansea University Medical School, UK; 2 Morriston Hospital, UK

**Keywords:** registries, registry data, natural language processing, research, clinical coding, medical record system, automated, health information management, cancer registries, computer assisted coding, automated coding

## Abstract

**Background:**

Automated clinical coding can use statistical or artificial intelligence-based technology to transform unstructured clinical data into clinical codes. These processes have the potential to enhance the quality and accuracy of data collections, save resources and accelerate research.

**Objective:**

To evaluate the use of automated clinical coding in the United Kingdom (UK) and European cancer registries.

**Method:**

An online electronic survey was formulated to evaluate the use and user opinion of automation within cancer registries. The survey was distributed to members of the United Kingdom and Ireland Association of Cancer Registry and the European cancer registries. Data analysis was performed using Microsoft Excel 2015^®^ version 15.13.3 in order to summarise the results.

**Results:**

Twenty-three of the 117 cancer registries responded to the distributed survey; 15 (12.8%) cancer registries used automation within their registry, mainly in the form of natural language processing or machine learning. Most of the sampled registries (73.3%) used these technologies to automate data collection from pathology reports; 87% of respondents reported automation as efficient; and 26.1% reported improved data quality; 12 (52.1%) of cancer registries still manually checked all the automations; and 17 (74%) respondents believed that the algorithms for difficult tasks require further development.

**Conclusion:**

Various computer-based algorithms have been used for automated clinical coding in the UK and European cancer registries in the past few decades; however, to date there are no published data to validate its use. Further research and development of these technologies is needed to ensure external validity and maximise the potential use within other cancer registries globally.

**Implications for health information management practice:**

It is clear that while automation can be advantageous in areas of clinical coding, the role of the “human” (HIMs and clinical coders) in coding and classifying registry data, and in overseeing the transition, will be required for some time yet.

## Introduction

Cancer registries play a crucial role in collecting patient data and driving improvements in cancer care (Cancer Research UK, 2018; Forsea, 2016; Giusti et al., 2023). These registries provide essential information on cancer burden, new guidelines, and quality and standards of healthcare services (Abildgaard et al., 2023; Forsea, 2016; Giusti et al., 2023). While cancer registration is mandatory by law in most of the European cancer registries (Forsea, 2016), there is still some variability in this process in some registries, where informed consent is required to register a cancer patient (Siesling et al., 2015). Despite regulations around cancer registration, there is a lack of good quality cancer registration in Europe. A third of European countries are affected by this, with 85% of the world’s population lacking accurate cancer case reporting (Forsea, 2016). The quality of data collection varies across Europe, with the highest performance being recorded within Nordic cancer registries, where 80% of the registries collect data to report epidemiology or for screening evaluation. The lowest reporting rates have been recorded in Eastern European cancer registries, where only 30% of the registries collect data for clinical audits, clinical guidelines or screening evaluation (Forsea, 2016; Siesling et al., 2015). Incomplete cancer data registration makes it challenging to estimate the true burden of disease (Ferlay et al., 2018), affecting cancer care, epidemiological research, public health planning and policy-making efforts. Furthermore, incomplete registration affects reporting rates of common as well as rare cancers, leading to underestimation of the true incidence of these cancers (Ferlay et al., 2018; Trama et al., 2016).

One such example of underreported cancer is basal cell carcinoma (BCC; Pukkala et al., 2018), the most common skin cancer worldwide (Gancan, 2022). BCC remains underreported in many cancer registries (Pukkala et al., 2018), where only the first occurrence of BCC or squamous cell carcinoma (SCC) is registered per patient lifetime (Cancer Research UK, 2014; Goodwin et al., 2004; National Cancer Registration and Analysis Service’s Cancer (NCRAS), 2022). This underreporting leads to a significant underestimation of the disease. For example, in the United Kingdom, true incidence is reported to be 50% higher (Goodwin et al., 2004; Ibrahim et al., 2023; Kwiatkowska et al., 2021). An exception to the SCC registration is Scotland, where registry staff manually register all of their cases, leading to a more accurate reporting of this type of cancer (Kwiatkowska et al., 2021; Venables et al., 2019). Routine data are collected from a variety of sources, including hospital records, discharge summaries, radiology departments, death certificates (Forsea, 2016), multidisciplinary team meetings, pathology reports, treatment records and molecular testing results (Henson et al., 2020). Although a variety of data sources are used to report cancer, there are shortfalls attributed to the lack of general population coverage by the cancer registries. Underdevelopment of computer-assisted coding systems in some European countries that hinder accurate reporting of cancer data is also thought to have a contributory role (Diz et al., 2017).

**Figure 1.**
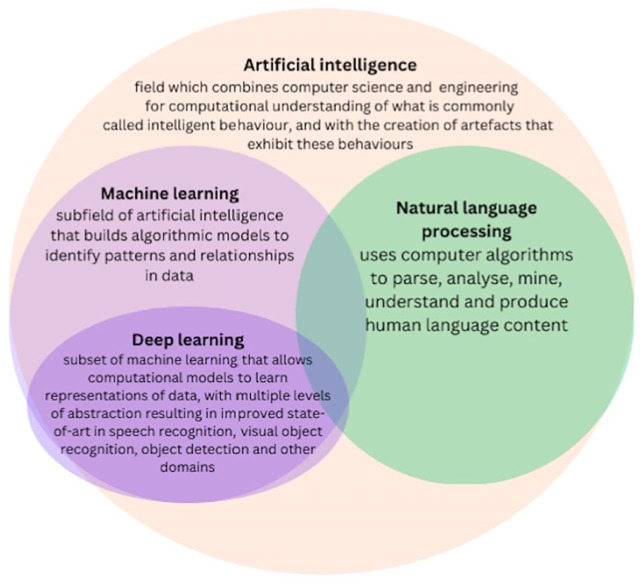
Diagram representing artificial intelligence algorithms commonly used for automation.

The European Network of Cancer Registries (ENCR, 2025) was developed in 1990 to facilitate collaborations between existing cancer registries and to improve cancer data collection and reporting (Forsea, 2016; Risk et al., 2018). One nascent method of improving data management is through automated clinical coding, which is a branch of computer-assisted coding that facilitates the translation of diagnostic unstructured data into a structured text, without human intervention (Dong et al., 2022; Stanfill et al., 2010). Artificial intelligence (AI) systems, which include several different types of algorithms (see Figure 1), have been widely used in cancer research (Kourou et al., 2015) to provide an evidence-based approach for a variety of tumours (Musa et al., 2022). AI methods, such as machine learning (ML) and deep learning (DL), have been used for cancer identification, screening, treatment and surveillance (Kalaiyarasi et al. 2020; National Cancer Institute Artificial Intelligence (AI) in Cancer Research, n.d.) and there is a growing trend in using these technologies to predict cancer outcomes, susceptibility, recurrence and survival (Kourou et al., 2015). DL, a subset of ML, has shown promise in improving cancer screening, treatment and surveillance (National Cancer Institute Artificial Intelligence (AI) in Cancer Research, n.d.).

Natural language processing (NLP) methods, which have been developed since the 1940s (Kimia et al., 2015), have the potential to advance oncological research, particularly in identifying cancer cases (Yim et al., 2016). These systems have also been successfully implemented in other domains such as patient prognosis prediction, interpretation of genomic data, detection of bacterial infections, discovery of novel biomarkers, radiology report recommendations, multiple sclerosis traits and the automation of adverse drug reaction detection (Kourou et al., 2021; Yim et al., 2016). Overall, these technologies have the potential to significantly improve cancer data collection and reporting, leading to better cancer care and improved public health outcomes. There are a variety of classification systems used within the European cancer registries, including International Classification of Diseases 9 (ICD-9), International Classification of Diseases 10 (ICD-10) and International Classification of Diseases O (ICD-O), alongside specialised systems for coding variables such as TNM staging, biomarkers or immunophenotype (Trojanowski et al., 2025). ICD-10 and Systematized Nomenclature of Medicine – Clinical Terms are used in some of the cancer registries, where the automation process is not fully developed yet. These computer-based technologies commonly use rule-based methods to encode free texts into machine-readable codes. However, the process can be automated with the aid of DL methods or NLP when applied to large datasets, reducing the labour-intensive work (Chen et al., 2021; Gaudet-Blavignac et al., 2021).

Automation has been trialed for use in clinical coding since the late 1960s, with its use continuing to grow in the healthcare industry (Dinwoodie and Howell, 1973 in Stanfill et al., 2010). In recent years, the quality and improvement in data collection, incorporating systems for staging of disease or ethnicity, has improved reporting of statistics on disease (Henson et al., 2020). Auto-processing of skin cancer, which involves automatic registration of BCC and SCC following extraction from pathology reports and nationalisation of cancer registration, has resulted in accuracy comparable to manual data extraction (Kwiatkowska et al., 2021). Implementation of computer-assisted coding has led to an improvement in the clinical coding accuracy, reducing the errors generated by manual coding and increasing the quality of data extracted (Campbell and Giadresco, 2020). Performance of the automation technologies depend on the complexity of the database used, with accuracy reaching 95% for certain extraction tasks (Nguyen et al., 2015). Automated clinical coding has shown promising results in extracting data from pathology reports within the cancer registries (Fabacher et al., 2020). Pathology reports tend to be more structured; therefore, NLP models generally perform well, with accuracy comparable to human extractors (Yu et al., 2021). Increasing implementation of automated clinical coding could facilitate clinical coders to focus more on ensuring completeness of the data for patients with missing information (Fabacher et al., 2020).

Despite the previous work of our group highlighting that the United Kingdom and Ireland Association of Cancer Registries (UKIACR) members all use some form of automated reporting, to date there is no literature examining specific methods of automated coding within the UK or European cancer registries (Ibrahim et al., 2021). Automation technologies have been successfully used to detect cancer cases from the electronic health records (EHRs), but their role within cancer registries needs to be quantified. The aim of this study was to establish the current use of automation in cancer registries in order to evaluate the role in cancer data collection.

## Method

### Materials and data collection

An online electronic questionnaire was designed using Google Forms (Google LLC, Menlo Park, CA, USA; see Appendix 1, Supplemental file) by clinicians in our research group with expertise in NLP, to assess current practice and harness opinion on the use of automation within UK and European cancer registries. The questions were specifically targeted towards the use of automated clinical coding, in order to establish the extent, type, common automated tasks and human intervention incorporated into current cancer registry practice. This was a multichoice questionnaire where respondents could select all the answers applicable to their cancer registry. Where answers were not applicable, respondents could reply as a free-text, represented as “Other” in the survey. Likert scales (1–5) were also used for some questions, ranked 1 (*strongly disagree*) to 5 (*strongly agree*). The survey was sent to all UKIACR members (NCRAS, Welsh Cancer Intelligence and Surveillance Unit, Scottish Cancer Registry, Northern Ireland Cancer Registry and National Cancer Registry Ireland) and to all European cancer registries that were publicly contactable on the ENCR website (European Network of Cancer Registries (ENCR) [2025]). We ran the survey from August to December 2022, with three follow-up emails sent until the deadline.

### Data analysis

Data analysis was performed using Microsoft Excel 2015^®^ version 15.13.3 (Microsoft Cooperation, Redmond, WA, USA). We tailored the survey to gain responses from those registry respondents who currently used automated clinical coding, and those that did not, in an effort to gauge broad opinions and perceptions on the technology.

### Ethics approval

Ethical approval was obtained from Swansea University Medical School, Wales, UK, prior to the start of the research study (SUMS RESC 2022-0059).

## Results

### Characteristics of participating registries

**Figure 2.**
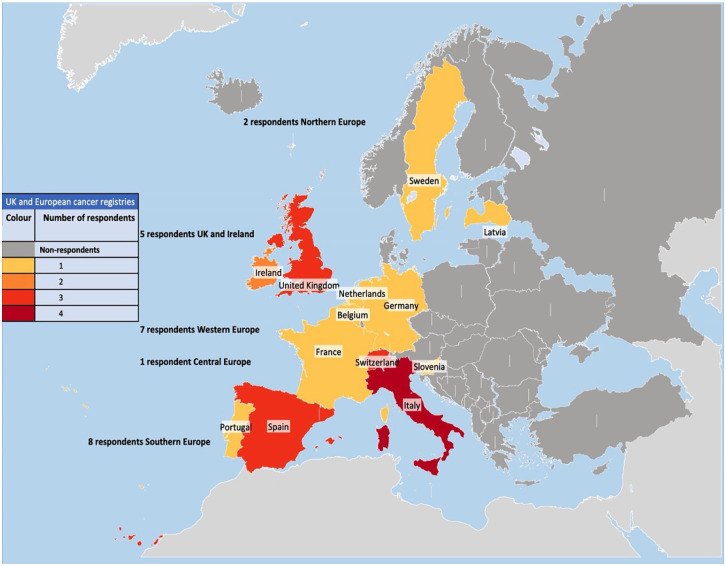
Heat map showing the UK and European cancer registries that replied to our survey with different colours representing the number of respondent cancer registries within each country.

Online questionnaires were sent to 117 cancer registries, representing 33 countries, in order to assess the variability in their use of automated clinical coding. In total, 23 responses were received from representatives of registries in the United Kingdom and Europe, giving an overall response rate of 19.6%. Representatives of all of the UK cancer registries (100%) and 18 of the European cancer registries (16.1%) responded. Participating European cancer registries were represented by Latvia and Sweden in Northern Europe; France, Netherlands, Belgium, Germany and Switzerland in Western Europe; Italy, Spain and Portugal in Southern Europe and Slovenia in Central Europe (see Figure 2); there were no responses from registries in Eastern Europe. Registry respondents to the survey represented a mix of cancer registry leads, statisticians, data analysts, data coordinators, data managers and registry managers (hereafter registry respondents).

### Automation inception and technology development

Of the participating cancer registries, 12 of 15 (80%) confirmed they used automation both in cancer care and skin cancer, with Latvia, Canaries and Madrid (20%) not having introduced any forms of automated coding for skin cancer at the time of the study. There was variability in the introduction of automated clinical coding within the cancer registries, with the earliest being introduced in the Northern Ireland Cancer Registry in January 1994. The earliest European cancer registry to introduce this technology was the Belgian Cancer Registry in January 2006, with the majority of registries using automation since 2015. These technologies were developed in-house in seven of the cancer registries (46.6%), in partnership with industry in three cancer registries (20%), and in partnership with academia in one cancer registry (6.6%). The Northern Ireland Cancer Registry and NCRAS developed automation tools in-house, in partnership with industry and academia, respectively.

The most common forms of automation used within the cancer registries were NLP and ML techniques, with some other novel forms of automation, as shown in Table 1. Medical record linkage used by the Scottish Cancer registry, involved matching patient records from various datasets in order to build health records, improve the quality of the health data, and to allow retrospective or prospective studies to be carried out (Coeli et al., 2015; Sauleau et al., 2005).

**Table 1.** Different types of automation used by the United Kingdom and European cancer registries.

Country/region	Cancer registry	Forms of automation
Scotland	Scottish cancer registry	MRL along with Oracle
Ireland	Northern Ireland cancer registry	In built system patient and tumour matching rules
	National cancer registry Ireland	None
England	National cancer registration and analysis service	NLP, ML, various lookup and mapping tables, other heuristics
Wales	Welsh cancer intelligence and surveillance unit	NLP
Sweden	Swedish cancer registry	None
Latvia	Latvian cancer registry	Uses automation, no response to forms of automation used
France	Haut-Rhin cancer registry	None
Netherlands	Netherlands cancer registry	Uses automation, no response to forms of automation used
Belgium	Belgian cancer registry	NLP
Germany	Cancer registry of North Rhine-Westphalia	NLP ML
Switzerland	Basel cancer registry	None
	Cancer registry of central Switzerland	ML, regular expressions
	Aargau cancer registry foundation	None
Italy	Cancer registry of Puglia-Province of Barletta-Andria-Trani	NLP
	Mantoya and Cremona cancer registry	None
	Veneto cancer registry	NLP
	Puglia cancer registry	NLP
Portugal	North region cancer registry of Portugal	None
Spain	Registro Poblacional de cancer de la comunidad autonoma de Canarias	NLP, SNOMED CT coding in pathological reports, ICD-10 in hospital discharge and death certificates
	Registro de cancer de Granada	None
	Registro Poblacional de cancer en la infancia y adolescencia de la comunidad de Madrid	NLP
Slovenia	Cancer registry of Republic of Slovenia	Uses automation, no response to forms of automation used

NLP: natural language processing; ML: machine learning; MRL: medical record linkage; ICD-10: international classification of diseases 10th revision; SNOMED-CT: systematized nomenclature of medicine-clinical terms.

### Data sources, common tasks used and perceived benefits of automation

**Figure 3.**
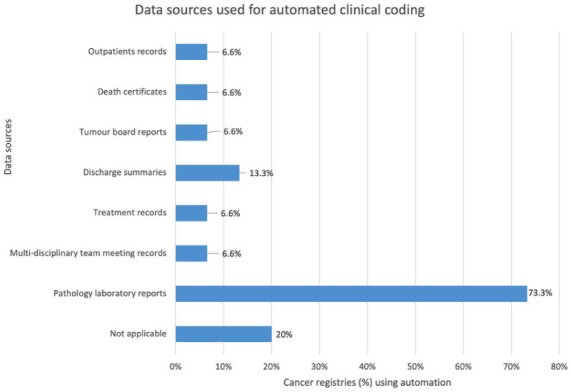
Bar chart demonstrating % out of the 15 cancer registries involved in automation and common data sources used within the cancer registries.

**Figure 4.**
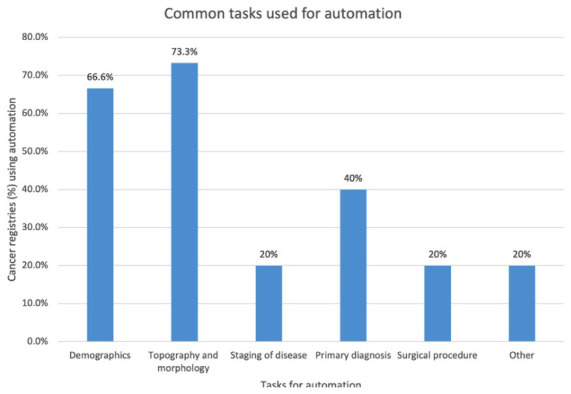
Bar chart showing % out of the 15 cancer registries using automation and the common tasks used within their registries. *Note*: Other: comorbidity, identification of new patients, extraction of biomarkers from histopathology reports.

The most common data sources used by the cancer registries were pathology laboratory reports in 11 of 15 cancer registries (73.3%), followed by discharge summaries in 2 cancer registries (13.3%), treatment records in 1 cancer registry (6.6%), multidisciplinary meeting records in 1 cancer registry (6.6%), tumour board reports in 1 cancer registry (6.6%), death certificates in 1 cancer registry (6.6%) and outpatients records in 1 cancer registry (6.6%; see Figure 3). Automated coding was mainly used for tasks in topography and morphology in 11 of 15 cancer registries (73.3%), demographics such as age, race, sex and postcode in 10 cancer registries (66.6%), primary diagnosis in 6 cancer registries (40%), staging of disease and surgical procedures in 3 cancer registries, respectively (20%) and comorbidities, identification of new patients or extraction of biomarkers from histopathology reports in 1 cancer registry, respectively (6.6%; see Figure 4).

Of the sampled cancer registries using automation, 9 (60%) considered this process best suited for automating data collection for pathological diagnosis; 8 for patient demographics (53.3%), 5 for epidemiological data (33.3%), 2 for comorbidities (13.3%), and 1 for tumour or patient matching, analysis of collected data or extraction of TNM staging and biomarkers from histopathology reports, respectively (6.66%). On the other hand, 8 of the 15 cancer registries (53.3%) reported that automation was least suited for surgical complications, 6 for patient comorbidities (40%), 3 for epidemiology data (20%), 2 for pathological diagnosis (13.3%) and 1 cancer registry (6.6%) for patient demographics, coding for high-quality data, topography and morphology and staging of disease, respectively. Interestingly, 6 of the 8 cancer registries (75%) that were not involved in automation, reported that these technologies were better suited for epidemiological data; and 5 (62.5%) suggested it was better suited for patient demographics, patient comorbidities or pathological diagnosis. Automation tasks were perceived as least suited for surgical complications in 4 out of 8 cancer registries (50%), patient demographics in 2 cancer registries (25%), comorbidities in 2 cancer registries (25%) and pathological diagnosis in 2 cancer registries (25%).

**Figure 5.**
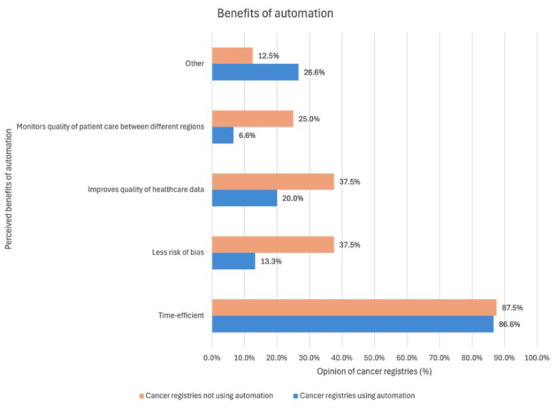
Clustered bar chart illustrating the opinion of the 15 cancer registries using automation and 8 cancer registries not currently involved in automation (described in %) and areas of perceived benefit within their cancer registry. *Note*: Other: Cancer registries using automation: helps improve data quality but it does not replace human coders’ work; only uses person data-place of living, sex; to get completeness of data; believe automation results in less quality information. Cancer registries not currently involved in automation: opens up potentially underutilised data sources, existing sources do not allow to be prioritised.

Of the 15 cancer registries using automation, 13 (86.6%) considered it to be time efficient; 3 (20%) that it improved the quality of healthcare data; 2 (13.3%) that it provided less risk of bias; and 1 cancer registry (6.6%) that it monitored the quality of patient care between different regions (see Figure 5). Individual cancer registries indicated that automation can create new provisional records, help to improve data quality, but does not replace human coders’ work. On the other hand, some registry respondents suggested that automation has the potential to automate data from underutilised data sources, although this process may result in information of poorer quality.

### Current performance and usage

Regarding current algorithms used in difficult automated tasks, 9 out of 23 registry respondents (39.1%) *strongly agreed* that it needed further development, 8 *agreed* (34.8%), 4 were *neutral* (17.4%) and 1 disagreed (4.3%); and 1 did not provide any answer to this question. Of the 15 cancer registries using automation, 12 registry respondents (80%) confirmed that humans were involved in this process to ensure good quality data collection. Only 1 out of 15 (6.6%) confirmed that they used automation for all the cancer registrations, while some cancer registries only used automation for certain tasks or certain tumour types.

## Discussion

The results of this study provide insight into the current use and perceptions of automated clinical coding in cancer registries across Europe, highlighting the variability in implementation, data sources used and perceived benefits of automation. While there was variability in the use of automated clinical coding in Europe, the majority of the UK cancer registries have already implemented automation in cancer care. The most commonly used forms of automation were NLP and ML techniques. This study also found that the most commonly used data sources were pathology laboratory reports, and the most common automated tasks were topography and morphology.

Cancer registration in the United Kingdom has been shown to be less accurate (McConnell et al., 2017). Completeness of cancer case identification depends on the quality of data sources used and reporting standards within the cancer registries (Merriel et al., 2017). Single data sources used within the UK cancer registries, such as death certificates, have been shown to miss up to 15% of cancer cases. This could be attributed to incorrect registration due to inexperienced clinicians or clinical coding errors (Kalsi et al., 2021). In England, data provided to the cancer registries are delivered from different sources. Therefore, manual verification with the assistance of automated tools has to be performed in order to ensure accuracy of data collection (Henson et al., 2020).

ML techniques have shown good performance on single cancer pathology reports classification in French cancer registries (Fabacher et al., 2020). ML has also been successfully used for the registration of both topography and morphology for multiple primary cancers, despite the variability in reporting pathology reports (Jouhet et al., 2012). These computer-based algorithms have been shown to improve cancer data linkage in order to match tumour records to patients in German cancer registries (Rochner and Rothlauf, 2024). The use of multiple sources such as operating notes, clinical letters or multidisciplinary team reports could increase the completeness of cancer data registration (Jouhet et al., 2012). Automating data extraction could reduce the time of data extraction up to a half compared to manual data extraction, allowing cancer registrars to focus more fully on ensuring completeness of clinical records (Fabacher et al., 2020).

Manual coding in cancer registries is a costly and time-consuming process, with up to 15% of full-time equivalent budgets being allocated towards it (HLA-Global, 2021). Furthermore, manual coding is prone to errors, and limitations on the volume and type of information collected can hinder accurate data collection (Kourou et al., 2021). Training clinical coders can also be a challenging process, requiring several months of education and ongoing training to stay up-to-date with changing standards (Dong et al., 2022). To address these issues, AI-based tools such as NLP have the potential to enhance the efficiency and quality of data extraction from EHRs, leading to near real-time cancer reporting (Mellia et al., 2021). NLP is a less expensive and more objective method of converting unstructured clinical text into usable data, and has the potential to create large datasets from EHRs, with the potential to improve cancer registry data capture and reporting (Mellia et al., 2021). The development and implementation of automated clinical coding tools should be a priority for cancer registries worldwide.

While cancer registry respondents to this study reported that automation was more time-efficient and improved the quality of healthcare data compared to human coding, there was still some reluctance to use these technologies without human involvement (Stanfill et al., 2010). Despite their efficiency, automation technologies are not without limitations, with poor accuracy observed when multiple parameters are involved (Stanfill et al., 2010). Of all registry respondents to our survey, 39.1% suggested that current automation technology requires further development in order to be used in complex tasks. Thus, while automation has proven to be as efficient as manual clinical coding in simple binary tasks (Stanfill et al., 2010), improvements in technology and further research are needed to enable its use in more complex tasks.

The use of NLP and other forms of automation in clinical coding has shown promising results, but limited external validation and a lack of standardised performance requirements have hindered widespread adoption in clinical and research settings (Burger et al., 2016; Mellia et al., 2021). Additionally, specific contextual and vocabulary terms present challenges in accurate coding, further highlighting the need for ongoing development and improvement of these tools (Kreimeyer et al., 2017). Limitations to the widespread adoption of these computer-based algorithms such as regulations, ability to integrate, and ethical factors will need to addressed in order to maximise the potential of these technologies (Ball, 2021). Despite these limitations, the potential benefits of automated clinical coding, including improved efficiency, reduced errors, and enhanced data extraction, make continued investment in these technologies a priority. Developing gold-standard clinical coding datasets and training models to adapt to changing terminologies will be crucial to improving the reliability and accuracy of these tools (Dong et al., 2022). In addition, regular validation and publication of outcomes by cancer registries involved in automation will promote wider adoption and knowledge-sharing among the medical community. Ultimately, improved cancer data capture and analysis through automation has the potential to directly improve patient care and outcomes.

### Strength and limitations

This study achieved a satisfactory response rate of 100% coverage in the United Kingdom, but only 16.1% coverage across mainland Europe, which may be attributed to the inability to contact all cancer registries within the ENCR. Only two countries in the Eastern and South-Eastern Europe with publicly contactable details on the ENCR website were contacted, but neither replied. Another limitation to this study was that the survey was designed in English, which could have represented a barrier for the Slavic language-speaking population. Also, certain aspects related to reporting pathways, funding resources, legislation regarding cancer data registration were not explored in this study. As there are currently no guidelines in place, we have contacted ENCR for recommendations on the use of automation, which is an area of interest they will need to explore in the future. This study found that the majority of respondents already used automation in their registries, potentially introducing a response bias as non-automated registries may have been less likely to respond. To address this bias, a separate survey was designed to capture the views of non-automated registries on the use of automated clinical coding. Nonetheless, these findings may underrepresent the prevalence of automated clinical coding use in Europe.

## Conclusion

In past decades, computer-based algorithms have been used for automated clinical coding in the UK and European cancer registries in different forms; however, to the best of our knowledge, there is no current literature underpinning the specific methods and role of automation within these registries. Our survey has shown that pathology reports were the main data source used to automate cancer data extraction in the cancer registries; therefore, standardised datasets and accurate reporting could leverage a wider application of automated clinical coding. This transition to full automation requires ongoing monitoring and evaluation to address any issues that may arise. Implementing automated clinical coding in health care could improve data quality and accuracy, save resources and accelerate research. Within the cancer registries, rigorous training of these computer-based algorithms is needed in order to overcome challenges to performing certain tasks and to maximise their potential in extracting data in order to improve cancer case capture. Our study was the first systematic investigation into the usage of automation within UK and European cancer registries, highlighting the underutilised potential of coding unstructured clinical data from EHRs. By delivering quantifiable and reportable results, automation has the potential to transform cancer research and patient outcomes.

## Supplemental Material

sj-docx-1-him-10.1177_18333583251378962 – Supplemental material for Automated data collection in cancer care: State of play among registries in the United Kingdom and Europe Supplemental material, sj-docx-1-him-10.1177_18333583251378962 for Automated data collection in cancer care: State of play among registries in the United Kingdom and Europe by Manuela Roman, Stephen Ali, Nader Ibrahim, Thomas D Dobbs, Hayley Hutchings and Iain S Whitaker in Health Information Management Journal
