# Supplementary material for: Automated data collection in cancer care: State of play among registries in the United Kingdom and Europe
Source: Health Inf Manag. 2025 Oct 7;55(1):123–31. doi: 10.1177/18333583251378962 (PMC12756524; doi:10.1177/18333583251378962)
Supplement: sj-docx-1-him-10.1177_18333583251378962 – Supplemental material for Automated data collection in cancer care: State of play among registries in the United Kingdom and Europe [file sj-docx-1-him-10.1177_18333583251378962.docx]

**Appendix 1: Survey template**

What is your professional role at your cancer registry e.g. statistician?

- Free text

Which cancer registry do you work at?

- England
- Scotland
- Northern Ireland
- Republic of Ireland
- Wales
- Northern Europe Cancer registry
- Southern Europe Cancer registry
- Eastern Europe Cancer registry
- Western Europe Cancer registry

1. Does your cancer registry use any form of automation during data collection, processing or analysis in cancer care?

- Yes
- No

**If answered “Yes” to question 1, please complete questions 2-13**

2. Does your cancer registry use automation for skin cancer data collection, processing or analysis?

- Yes
- No

3. If yes, what year was this introduced

MM/DD/YYYY

4. What tasks is automation used for?

- Age, race, sex, postcode/location
- Topography and morphology (location of cancer and subtype)
- Staging of disease
- Primary diagnosis
- Surgical procedure
- Not applicable
- Other

5. What tasks in data collection, processing or analysis is automation BETTER suited to at your cancer registry?

- Patient demographics
- Epidemiology data e.g. incidence, prevalence, mortality
- Patient comorbidities
- Pathological diagnosis
- Surgical complications
- Not applicable
- Other

6. What tasks in data collection, processing or analysis is automation LEAST suited to at your cancer registry?

- Patient demographics
- Epidemiology data e.g. incidence, prevalence, mortality
- Patient comorbidities
- Pathological diagnosis
- Surgical complications
- Not applicable
- Other

7. What forms of automation do you use at your registry?

- Natural language processing
- Machine learning
- Deep learning
- Combinations of all of the above
- None
- Other

8. Are there any specific data source(s) where automated clinical coding is used at your cancer registry?

- Diagnostic imaging reports
- Pathology laboratory reports
- Treatment records
- Multi-disciplinary team (MDT) meeting records
- Clinical cancer audits
- Not applicable
- Other

9. How was this technology developed at your registry?

- In partnership with academia
- In partnership with industry
- Developed in-house
- Not applicable

10. What proportion of cancer registrations at your registry use these technologies?

- %
- Do not know
- Other

11. What are your perceived benefits of automation?

- It is time-efficient
- Less risk of bias
- Improves quality of healthcare data
- Monitors quality of patient care between different regions
- Other

12. Do your feel current algorithms need to be further developed in order to improve performance of difficult tasks in automation?

- Likert scale 1-5

13. Is there also a human intervention for quality assurance within automated clinical coding e.g. "human-in-the-loop"?

- Yes
- No
- Do not know

**If answered "No" to question 1, please complete questions 14-20**

14. What tasks do you feel automation may be better suited to if adopted at your cancer registry?

- Age, race, sex, postcode/location
- Topography and morphology (location of cancer and subtype)
- Staging of disease
- Primary diagnosis
- Surgical procedure
- Not applicable
- Other

15. What tasks in data collection, processing or analysis do you feel automation may be BETTER suited to if adopted at your cancer registry?

- Patient demographics
- Epidemiology data e.g. Incidence, prevalence, mortality
- Patient comorbidities
- Pathological diagnosis
- Surgical complications
- Not applicable
- Other

16. What tasks in data collection, processing or analysis do you feel automation is LEAST suited if adopted at your cancer registry?

- Patient demographics
- Epidemiology data e.g. Incidence, prevalence, mortality
- Patient comorbidities
- Pathological diagnosis
- Surgical complications
- Not applicable
- Other

17. What forms of automation are you familiar with?

- Natural language processing
- Machine learning
- Deep learning
- None
- Other

18. What proportion of cancer registrations at your registry do you think that the above technologies could potentially be used on for purposes of data collection, processing or analysis?

- (%)
- Do not know
- Other

19. What are the perceived benefits of automation, in your opinion?

- It is time-efficient
- Less risk of bias
- Improves quality of healthcare data
- Monitors quality of patient care between different regions
- Other

20. Do you feel that the performance of current artificial intelligence algorithms (machine learning, natural language processing or deep learning) need to be developed further before they can be used routinely at your cancer registry?

- Likert scale 1-5

Other: free-text; where applicable, respondents could select all the answers that apply to their cancer registry.
